# The hTERT and iCasp9 Transgenes Affect EOMES and T-BET Levels in NK Cells and the Introduction of Both Genes Improves NK Cell Proliferation in Response to IL2 and IL15 Stimulation

**DOI:** 10.3390/biomedicines12030650

**Published:** 2024-03-14

**Authors:** Anastasia I. Palamarchuk, Elena I. Kovalenko, Maria A. Streltsova

**Affiliations:** Shemyakin & Ovchinnikov Institute of Bioorganic Chemistry, Russian Academy of Sciences, Ul. Miklukho-Maklaya 16/10, 117997 Moscow, Russia; palanastasia@yandex.ru (A.I.P.); lenkovalen@mail.ru (E.I.K.)

**Keywords:** NK cells, EOMES, T-BET, *hTERT*, *iCasp9*, stimulation, exhaustion, immune checkpoints, survival

## Abstract

The NK cell exhaustion state evolving during extensive and prolonged cultivation is still one of the limitations of NK cell approaches. In this research, we transduced NK cells with the *hTERT* and *iCasp9* genes. hTERT overexpression can prevent the functional exhaustion of NK cells during long-term cultivation, but, still, the therapeutic use of such cells is unsafe without irradiation. To overcome this obstacle, we additionally transduced NK cells with the *iCasp9* transgene that enables the rapid elimination of modified cells. We compared the proliferative and functional activities of the hTERT- and/or iCasp9-modified NK cells, determined their exhaustion state and monitored the levels of EOMES and T-BET, the main NK cell transcription factors. The *hTERT* and *iCasp9* genes were shown to affect the EOMES and T-BET levels differently in the NK cells. The EOMES^+^T-BET^+^ phenotype characterized the functionally active NK cells during two months of culture upon stimulation with IL2 and K562-mbIL21 feeder cells, which induced the greatest expansion rates of the NK cells, independently of the transgene type. On the other hand, under cytokine stimulation, the hTERT-iCasp9-NK cells displayed improved proliferation over NK cells modified with iCasp9 alone and showed an increased proliferation rate compared to the untransduced NK cells under stimulation with IL2 and IL15, which was accompanied by reduced immune checkpoint molecule expression. The individual changes in the EOMES and T-BET levels strictly corresponded to the NK cell functional activity, the surface levels of activating and inhibitory receptors along with the expansion rate and expression levels of pro-survival and pro-apoptotic genes.

## 1. Introduction

NK-cell-based therapeutic approaches for cancer therapy are rapidly evolving due to NK cells’ innate ability to recognize and eliminate malignant cells without prior sensitization. To increase their therapeutic utility, NK cells are often subjected to genetic modifications using retroviral transduction aimed at enhancing their functional activity and target recognition. These modifications require time between the ex vivo isolation of NK cells and their ‘ready to use’ state. However, one of the problems of the prolonged in vitro cultivation before and after NK cell modification may be the reduction in their overall functionality and proliferative activity at the moment of therapeutic use.

Sustained cytokine stimulation during long-term cultivation in vitro may lead to NK cell exhaustion [1]. The exhausted cells lose the ability for intensive functional activity like IFNγ production or cytotoxicity towards target cells. Phenotypically, NK cell exhaustion is associated with decreased expression of a set of activating surface molecules [2], while immune checkpoint molecules (PD-1, TIM-3, LAG-3, TIGIT) increase their expression [3]. The KLRG-1 surface molecule also defines terminally differentiated and exhausted NK cells, which exhibit a low proliferative and functional response [1,4].

The functional state of NK cells, especially at the late differentiation stages, is closely related to the EOMES and T-BET transcription factors. The EOMES and T-BET gene targets overlap. Namely, EOMES and T-BET regulate the expression of cytokine receptors, factors associated with the apoptotic cascade and proliferative activity. The stepwise change in the predominant expression of EOMES to T-BET determines NK cell maturation. EOMES^+^ T-BET^+^ NK cells show greater functional activity, including IFNγ production and cytotoxicity, whereas EOMES^−^ T-BET^−^ NK cells show an exhaustion phenotype [5,6,7,8].

Cytokine stimulation and further cytokine support significantly affects the functional activity and duration of NK cell persistence in adoptive therapy [9]. In vitro and in vivo, IL2 and IL2+IL15 cytokines are widely used for NK cell stimulation. Transmitting signals via the IL2R/β heterodimers IL2 and IL15 improve the expansion, cytotoxicity and overall survival of NK cells and also cause the upregulation of *hTERT* expression [10,11,12,13,14,15]. Stimulation by these cytokines contributes to the maintenance and/or activation of various BCL2-family anti-apoptotic factors (Bcl-2, Bcl-X_L_ and Mcl-1) [16]. However, sustained cytokine signaling upregulates suppressors of cytokine signaling (SOCS) which inhibit Jak/STAT activation and signal transduction. The increased levels of SOCS, namely, SOCS-1, SOCS-2, SOCS-3 and Cis, upregulated by IL2 and IL15 impair signal transduction from cytokines and promote NK cell exhaustion by the regulation of a wide range of genes of NK cell differentiation, survival, proliferation, cytokine production and cytotoxicity [11]. Although cytokine priming has traditionally been used to activate NK cells, the highest expansion rates were obtained using feeder cell lines [17,18]. The preliminary activation of NK cells by a combination of IL2 and irradiated K562-mbIL21 cells increases the efficiency of retroviral transduction [19]. The use of the IL2+K562-mbIL21 stimulated cells improves the telomere length [20] and functional state of NK cells: activating proliferation, IFNγ production, cytotoxicity and increasing surface expression of CD86, NKG2D and HLA-DR [21].

To overcome possible NK cell exhaustion, transduction of the *hTERT* gene encoding the human telomerase reverse transcriptase catalytic subunit may be used. hTERT has already been shown to extend the replicative potential and activation state of NK cells cultured in vitro for months [22,23]. It was shown that even after a year of cultivation, NK cells maintained expression of the receptors responsible for their activation such as NKp30, NKp44, NKp46 and NKG2D [22]. hTERT also prevents cell death by various non-canonical pathways. For example, it participates in mitochondria maintenance and mediates the expression of a wide range of genes [24,25,26]. However, hTERT overexpression is normally associated with cancer transformation [27], so the utilization of such cells without irradiation or an appropriate control raises some safety concerns [28,29]. This problem may be resolved by the additional modification of therapeutic cells with a suicide gene construct such as the *iCasp9* suicide gene. iCasp9 is a synthetic construct consisting of a caspase 9-encoding sequence lacking CARD (delta caspase 9) bound to the FKBP domain responsible for the dimerization and subsequent activation of caspase 9. Chemical inductor of dimerization binds to FKBP and dimerizes the construct [30]. However, spontaneous dimerization may occur and lead to unintentional cell destruction [31].

In this work, we obtained three kinds of gene-engineered NK cells modified with pro-survival and pro-death genes: hTERT-NK cells, hTERT-iCasp9-NK cells and iCasp9-NK cells, and compared their proliferative capacity and viability after the long-term culture of the gene-engineered and untransduced cells in different stimulation conditions. To figure out if hTERT- and/or iCasp9-modified NK cells acquire any characteristics of exhaustion, we performed a series of experiments determining their proliferative and functional activity along with the expression of a set of genes encoding factors of the apoptotic cascade and surface markers of NK cell activation and exhaustion. We also traced the changes in the expression levels of EOMES and T-BET transcription factors known for the regulation of the main NK cell properties.

## 2. Materials and Methods

### 2.1. Experimental Design

**Figure 1:**
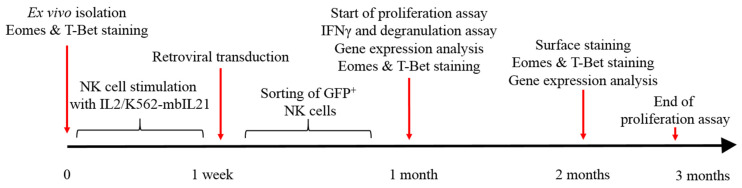
Experiment design on a timescale.

In this study, we explored the acquisition of functional exhaustion in NK cells modified with the *hTERT* gene and *iCasp9* gene. Data on the proliferative and functional activity of these cells were collected. The experimental design is presented in Figure 1. At starting point “0”, NK cells were isolated ex vivo, stained intracellularly with EOMES and T-BET and measured by flow cytometry. Then, freshly isolated NK cells were stimulated for a week with IL2 (100 U/mL) and K562-mbIL21 irradiated feeder cells at the ratio 1:1 NK cells:K562-mbIL21. At the 1 week time point, stimulated NK cells were transduced by retroviral particles bearing *iCasp9* and/or *hTERT* genes. Modified NK cells were separated from unmodified NK cells by the presence of GFP reporter in them. Cell sorting was performed. At the time point of 1 month, 4 subsets of NK cells (untransduced, hTERT-NK cells, hTERT-iCasp9-NK cells, iCasp9-NK cells) were studied for their proliferation (proliferation assay) and functional (IFNγ and degranulation assay) activity, gene expression and EOMES and T-BET protein levels. At the time point of 2 months, NK cells underwent surface and EOMES and T-BET staining. Gene expression levels were also evaluated. Around 3 months after ex vivo isolation of NK cells, the experiment was terminated due to the death of the majority of the NK cells studied.

### 2.2. Cell Lines

K562 cell line obtained from ATCC (Manassass, VA, USA) was cultured in RPMI medium (PanEco, Moscow, Russia) supplemented with 5% fetal calf serum (FCS, HyClone Labs, Logan, UT, USA), 2 mM alanine–glutamine (PanEco, Moscow, Russia) and 2 mM antibiotic–antimycotic (Sigma-Aldrich, St. Louis, MO, USA). K562 cells with membrane-bound IL21 (K562-mbIL21) kindly provided by Dr. D. Lee (MD Anderson Cancer Center, Houston, TX, USA) were cultivated in the same way as K562 cell line and γ-irradiated by 100 Gy using Varian Truebeam (Varian, Palo Alto, CA, USA) and were then utilized as feeder cells. Phoenix-Ampho cell line produced from HEK293T cell line modified for constitutive expression of *Gag-Pol*/*Tat*/*Env*/*Rev* viral genes was cultured in DMEM medium (PanEco, Moscow, Russia) supplemented with 10% FCS (HyClone Labs, Logan, UT, USA), 2 mM sodium pyruvate (PanEco, Moscow, Russia), 2 mM alanine-glutamine (PanEco, Moscow, Russia), and 2 mM antibiotic-antimycotic (Sigma-Aldrich). All cell cultures were cultivated in a CO_2_ incubator at 37 °C.

### 2.3. NK Cells Isolation and Stimulation Prior Transduction

Peripheral blood derived from healthy volunteers providing their informed consent approved by the local ethics committee was used for PBMC (peripheral blood mononuclear cell) isolation on a 1.077 g/mL Ficoll gradient (PanEco, Moscow, Russia). NK cells were isolated from PBMC using negative magnetic separation with an NK separation commercial kit (Miltenyi Biotec, Bergisch Gladbach, Germany) according to the manufacturer’s instructions. Freshly isolated NK cells were stimulated with 100 U/mL of IL2 (Hoffmann La-Roche, Basel, Switzerland) and γ-irradiated at 100 Gy along with K562-mbIL21 feeder cells at a ratio of 2/1 = NK cells/K562-mbIL21 for 4–6 days prior to transduction procedure. NK cells were cultured in NK cell medium mixed with 45% DMEM (PanEco) supplemented with 2 mM sodium pyruvate (PanEco), 2 mM alanine–glutamine (PanEco), 45% NK MACS medium with its supplement (Miltenyi Biotec), 10% FCS (HyClone Labs, Logan, UT, USA), and 2 mM antibiotic–antimycotic (Sigma-Aldrich). NK cell concentration was maintained from 6 × 10^5^ cells/mL up to 10^6^ cells/mL.

### 2.4. Assembly of Xlox TERT PGK iCasp9 IRES GFP Plasmid

Plasmids bearing the *hTERT* gene xlox(GFP)hTERT (Addgene #69809) and *iCasp9* gene pMSCV-F-del Casp9.IRES.GFP (Addgene #15567) were used to deliver *hTERT* and *iCasp9* genes into the cells. The plasmid xlox TERT PGK iCasp9 IRES GFP consisting of both genes was assembled from Addgene #69809 and Addgene #15567. Thus, both genes, *hTERT* and *iCasp9*, were simultaneously transduced to NK cells that exhibited resistance to genetic modification.

The sequence containing iCasp9-IRES-GFP was amplified by Encyclo polymerase (Evrogen, Moscow, Russia) and primers NotI iCasp9 F (5′-ctttcggcggccgcaatgctcgagggagtgcag-3′) and iCasp9 BFP R (5′-gcctgcaggtcgactctag-3′). Then, the restriction of a target plasmid xlox(GFP)hTERT (Addgene #69809) and PCR fragments was carried out by endonuclease NotI (isoshizomer CciNI, SibEnzyme, Novosibirsk, Russia) in SE-buffer Y (SibEnzyme). Fragments were purified with the Cleanup Mini kit (Evrogen) on each step according to the manufacturer’s instructions.

In order to avoid self-closure of the plasmid fragment at the NotI site as a result of ligation, it was pretreated with Shrimp Alkaline Phosphatase (rSAP) (New England Biolabs, Ipswich, MA, USA) in the rCutSmart™ Buffer (New England Biolabs), which removes phosphate groups from the 5′ ends. The fragment treated with phosphatase was cleaned with a Cleanup Mini kit (Evrogen) and then mixed for subsequent ligation with T4 DNA Ligase (SibEnzyme) in a 5× Quick Ligation Buffer (SibEnzyme) for 15 min according to the manufacturer’s recommendations.

Amplification of plasmid DNA was conducted in *E. coli XL1-Blue* bacteria cell line cultivated in selective LB+ampicillin medium. The plasmid was extracted by Plasmid Miniprep and Plasmid Midiprep 2.0 kits (Evrogen) in accordance with the manufacturer’s protocol.

The assembled plasmid was verified by Sanger sequencing provided by Evrogen (Russia) from primer iCasp9 R (5′-aagacgagagtggcatgtgg-3′).

DNA concentration was measured by Biodrop (Innovative Solutions, Carson, NV, USA) on each step.

### 2.5. Production of Retroviral Particles

Phoenix-Ampho cell line was transferred to poly-L-lysine (Sigma-Aldrich)-covered Petri dishes and then transfected using calcium phosphate transfection kit (Biospecifica, Novosibirsk, Russia) according to manufacturer’s instructions. RD114-bearing plasmid mixed in a ratio of ½ with target-gene-bearing plasmid was used for transfection. hTERT and iCasp9 genes were delivered by xlox(GFP)hTERT (Addgene #69809) and pMSCV-F-del Casp9.IRES.GFP (Addgene #15567) constructs, correspondingly. Viral supernatant was harvested from 24 h to 72 h post-transfection, filtered by Millex-HV-0.45 µm PES filter (Millipore, Burlington, NJ, USA) and concentrated via ultracentrifugation at 21,000× *g*, 4 °C, 2.5 h.

### 2.6. Retroviral Transduction

Transduction was performed in 24-well plates covered with 20 µg/mL Retronectin (Clontech/Takara, Terra Bella Ave., Mountain View, CA, USA) solution in PBS (PanEco). Concentrated viral particles were centrifuged at 1800× *g*, 2.5 h on retronectin, then removed and replaced with activated NK cells in NK cell medium at a cell density of 700,000 cells/mL and centrifuged (200× *g*, 45 min, 37 °C). Transduced NK cells were incubated in a CO_2_ incubator at 37 °C. On day 3, NK cells were removed from retronectin-covered wells.

### 2.7. NK Cell Sorting

Transduced NK cells were separated from untransduced NK cells according to the presence of GFP fluorescence using FACSVantageDiVa cell sorter (Becton Dickinson, Franklin Lakes, NJ, USA), equipped with 405, 488 and 643 nm lasers.

### 2.8. Cultivation of Transduced NK Cells

One month after ex vivo isolation, transduced and sorted NK cells and unmodified control NK cells were examined for proliferation, expansion and persistence in vitro. Three types of stimulation methods were examined in triplicate. First, NK cells were cultured in the presence of 100 U/mL of IL2. Second, NK cells were kept in a medium containing 100 U/mL of IL2 in combination with monthly added feeder K562-mbIL21 at a ratio of 2/1 = NK cells/K562-mbIL21. The last type of stimulation was performed using a combination of 100 U/mL of IL2 with 10 ng/mL of IL15 (Sigma-Aldrich). NK cells were cultured in 96-well U-bottom plates with an initial number of 75,000 cells per well. Half medium change was performed twice a week.

### 2.9. Flow Cytometry

Flow cytometry analysis was performed with the use of MACSQuant 10 cytometer (Miltenyi Biotec, Bergisch Gladbach, Germany) equipped with 405 nm, 488 nm and 635 nm lasers. Transduction efficiency was verified on day 5 after the transduction procedure by detection of GFP fluorescence. Surface staining with KLRG-1-APC (AntibodySystem, Schiltigheim, France, code: FHK277200-APC), TIM-3 (Biolegend, clone F38-2E2), TIGIT-PE (AntibodySystem, code: FHH72420-PE), PD-1-AF647 (Biolegend, clone: EH12.2H7), NKG2A-PE-Vio770 (Miltenyi Biotec, clone: REA1161), NKG2C-APC (R&D, cat: FAB138A), HLA-DR-PE-Vio770 (Miltenyi Biotec, clone: REA805), KIR2DL2/3-APC (Miltenyi Biotec, clone: DX27), NKp30-PE (Sony, clone: P30-15), NKp44-PE-CY7 (Sony, clone: P44-8), NKp46-AF647 (Sony, clone: 9E2), CD16-PE (Sony, clone: 3G8), CD56-APC-Vio770 (Miltenyi Biotec, clone: REA196), CD56-PE-Cyanine7 (Sony, clone:5.1H11), CD56-BV421 (Sony, clone5.1H11) CD57-APC (Sony, clone: HKN-1). Staining with antibodies was carried in a PBA buffer containing PBS, 0.5% BSA (Serva, Heidelberg, Germany) and 0.01% sodium azide (AMRESCO Inc., Aurora, CO, USA).

The expression of hTERT and EOMES and T-BET transcription factors was evaluated using a rabbit polyclonal antibody to TERT (Affinity Biosciences, San Francisco, CA, USA, cat#DF7129) with a secondary 647-conjugated goat anti-rabbit IgG (H+L) (ABclonal, Woburn, MA, USA, cat#AS060), EOMES-eFlour660 monoclonal antibody (clone: WD1928) (Invitrogen, San Jose, CA, USA) and T-BET-PE monoclonal antibody (clone: 4B10) (Biolegend, San Diego, CA, USA). Cells were fixed with eBioscience™ Foxp3/Transcription Factor Staining Buffer Set (Ref: 00-5523-00, Lot: 1945425) (Invitrogen) according to the manufacturer’s instructions.

### 2.10. Degranulation Assay

Unmodified and transduced NK cells were compared with a degranulation assay. CD107a (LAMP-1-PERCP (Sony, clone: H4A3) surface expression was detected after co-cultivation of NK cells with their targets (K562 cell line). For 24 h, NK cells were maintained in the culture medium without IL2 at a density of 1 mln cells/mL. Next, these cells were stimulated with 500 U/mL of IL2 overnight. Then, target K562 cells were added at the ratio 1/1 and incubated for 2.5 h, at 37 °C with CO_2_, in the presence of anti-CD107a mAb (CD107a-PE-Cy7, clone: H4A3 Sony Biotechnology, San Jose, CA, USA) and 10 µg/mL of Brefeldin A (Invitrogen) and in culture medium with IL2 (500 U/mL). Finally, flow cytometry analysis was performed. A mixed population of NK cells (untransduced GFP^−^ and transduced GFP^+^) was used for the degranulation assay and modified NK cells were determined by gating.

### 2.11. IFNγ Assay

IFNγ production response was measured among transduced and untransduced NK cells. Firstly, cells were maintained in the culture medium without IL2 at a density of 1 × 10^6^ cells/mL for 24 h. Secondly, NK cells were cultivated in the presence of IL2 (100 U/mL), IL18 (20 ng/mL) (R&D system, Lake Bluff, IL, USA) and IL12 (20 ng/mL) (R&D system, Lake Bluff, IL, USA) overnight and for 4 h with addition of 10 µg/mL of Brefeldin A (Invitrogen). Intracellular staining of IFNγ by Ab (IFNγ-PE (Miltenyi Biotec, clone REA600) was performed with the use of BD Cytofix/Cytoperm™ kit (BD Biosciences, San Jose, CA, USA) in accordance with the manufacturer’s protocol. A mixed population of NK cells (untransduced GFP^−^ and transduced GFP^+^) was used for IFNγ assay and modified NK cells were determined by gating during flow cytometry analysis.

### 2.12. Apoptosis Induction in iCasp9-NK Cells

Unmodified and iCasp9 cells were maintained for 24 h in cell medium with addition of PBS, DMSO or 100 nM of chemical inductor of dimerization (CID) AP20187 (MedChemExpress, Monmouth Junction, NJ 08852, USA). DMSO was used at a concentration corresponding to its content in a sample with 100 nM CID. Cell death induction rate was examined by AnnexinV-PE (Invitrogen, San Jose, CA, USA) and SYTOX AADvanced Dead Cell Stain Kit (Invitrogen, San Jose, CA, USA) staining according to the manufacturer’s instructions. Dead cells were determined as positive for AnnexinV and/or SYTOX staining. The increase in percentage of dead NK cells was calculated by the following formula: Δ% = 100 nM CID-treated dead NK cells % − dead NK cells in control %.

### 2.13. Cell Count

Equal volumes of cell suspension were picked in each measurement. NK cells were autolabeled with half volume of 1/1000 PBS solution on SYTOX-VioBlue (Invitrogen). Live cells were determined by both GFP fluorescence in modified cells and absence of SYTOX-VioBlue fluorescence.

### 2.14. QPCR Analysis

mRNA expression levels of genes *ACTB*, *BAD*, *BAK1*, *BAX*, *BCL2*, *BCL2L1*, *BIRC5*, *DIABLO*, *EOMES*, *iCASP9*, *MCL1*, *PMAIP1*, *BBC3*, *TBX21*, *hTERT*, *TIM3*, *TIGIT*, *PD1*, *LAG3*, *CISH*, *SOCS1*, *SOCS2*, *SOCS3* were investigated via qPCR (quantitative polymerase chain reaction) in transduced and sorted NK cells a month after ex vivo isolation. Total mRNA was extracted using ExtractRNA reagent (Evrogen) following the manufacturer’s recommendations. Genomic DNA was removed with TURBO DNA-free™ Kit (Invitrogen). cDNA was obtained by reverse transcription from the standard 18dT primer using MMLV reverse transcriptase (Evrogen). For qPCR, cDNA was amplified with a set of primers listed in Table S1. The assay was conducted in CFX Connect Real-Time System (Bio-Rad Laboratories, Hercules, CA, USA) using intercalating dye qPCRmix-HS SYBR (Evrogen) for signal detection.

### 2.15. Telomerase Test

The telomerase catalytic activity was tested by the telomeric repeat amplification protocol (TRAP) described in detail in [32]. The Q-TRAP method was used. Briefly, the Q-TRAP method is combined in two steps. First, telomerase reverse transcriptase from lysed cells elongate telomeric repeats. Second, forward and reverse primers amplify sequence produced due to telomerase activity. Amplification signal is measured using SYBR Green for detection and analysis with normalization on baseline signal.

### 2.16. Electronic Resources

Flow cytometry data were analyzed by FlowJo software version V10 (TreeStar Williamson Way, Ashland, OR, USA). Statistical analysis was carried out using GraphPad Prism 9.5 (StatSoft Inc., Tulsa, OK, USA) software. *p*-value: * *p* < 0.05; ** *p* < 0.01; *** *p* < 0.001. Primer selection was conducted using Primer BLAST (NCBI, Bethesda, MD, USA) web tool. The plasmid reassembly plan was carried out in the SnapGene v3.2.1. (GSL Biotech LLC, Boston, MA, USA) program. qPCR data were analyzed in the BioRad CFX Maestro program (Bio-Rad Laboratories, Hercules, CA, USA).

## 3. Results

### 3.1. Obtaining NK Cells Modified with hTERT and iCasp9 Genes 

**Figure 2:**
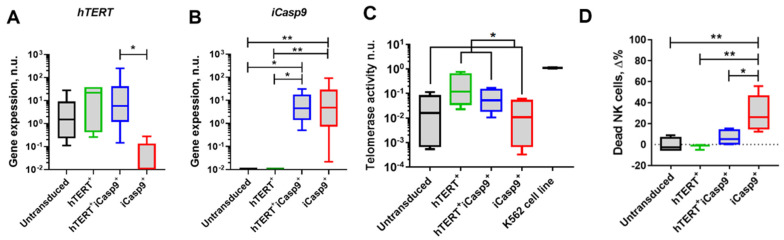
Retroviral transduction of NK cells with *hTERT* or/and *iCasp9* genes. (**A**) The *ACTB* normalized expression levels of *hTERT* transgene evaluated by qPCR analysis (*n* = 7). * *p* < 0.05. (**B**) The *ACTB* normalized expression levels of *iCasp9* transgene evaluated by qPCR analysis (*n* = 7). Friedman test with Dunn’s multiple comparison test, median, *p*-value: * *p* < 0.05; ** *p* < 0.01. (**C**) Telomerase activity in modified NK cells determined by TRAP assay for qPCR method (*n* = 4). The K562 cell line was used as positive control. For comparison, data were divided into 2 groups (1st with hTERT transgenes: hTERT^+^ with hTERT^+^iCasp9^+^; 2nd without *hTERT* transgenes: untransduced with iCasp9^+^). Mann–Whitney test, median, *p*-value: * *p* < 0.05. (**D**) Proportion of dead NK cells treated for 24 h with 100 nM chemical inductor of dimerization (CID) (*n* = 6). NK cells cultured without CID were used as a control. Dead cells were determined as positive for AnnexinV and/or SYTOX staining. The increase in percentage of dead NK cells was calculated by the following formula: Δ% = 100 nM CID-treated dead NK cells %—dead NK cells in control %. Ordinary one-way ANOVA with Tukey’s multiple comparison test, mean, *p*-value: * *p* < 0.05; ** *p* < 0.01.

The activated NK cells were transduced by retroviral particles bearing the *hTERT* and *iCasp9* genes. The expression levels of the transgenes were verified by qPCR on the NK cells sorted by the presence of GFP fluorescence. The *hTERT* expression level was higher in hTERT^+^ (median: 21.6) and hTERT^+^iCasp9^+^ (median: 5.9) NK cells compared with the untransduced (median: 1.5) and iCasp9^+^ (median: 0) NK cells (Figure 2A). *iCasp9* gene expression was detected in the hTERT^+^iCasp9^+^ (median: 4.6) NK cells and the Casp9^+^ (median: 4.8) NK cells, whereas in the hTERT^+^ and untransduced NK cells, iCasp9 expression was undetectable (Figure 2B). The enhanced hTERT catalytic activity (median = 0.1) was also observed among the NK cells modified with the corresponding gene compared to the untransduced and iCasp9^+^ NK cells (median = 0) (Figure 2C).

The efficiency of cell death induction in the iCasp9-modified NK cells was studied 24 h after the cell was exposed to a chemical inductor of dimerization (CID) (Figure 2D and Figure S1). The CID did not induce apoptosis in the hTERT-modified or unmodified NK cells, whereas (hTERT)-iCasp9-NK cells partly died in response to the dimerizer. The CID response level in the iCasp9-NK cells with hTERT overexpression was reduced relative to the iCasp9-NK cells without additional modifications (Figure 2D and Figure S1). Thus, the hTERT-iCasp9-NK cells were significantly more resistant to apoptosis induction by the dimerizer. We assumed that a decrease in the effectiveness of the CID-mediated apoptosis induction in the hTERT-iCasp9-NK cells may be associated with the overexpression of hTERT.

### 3.2. hTERT-iCasp9-NK Cells Are Characterized by Enhanced Proliferation and Survival under Different Conditions

To study how the insertion of the pro-survival *hTERT* and suicide switch *iCasp9* genes into NK cells affects their ability to proliferate over long-lasting cultivation, we tracked the cell number, expansion rate and the proportion of live cells for untransduced NK cells, hTERT-NK cells, hTERT-iCasp9-NK cells and iCasp9-NK cell cultures. The proliferation test for these cells was started a month after isolation. We used IL2 only and IL2+IL15 stimulations as conservative methods supporting NK cell survival, while a combination of IL2+K562-mbIL21 was applied in order to achieve abundant proliferation of transduced NK cells as a well-known type of stimulation inducing the effective expansion of NK cells [20]. 

**Figure 3:**
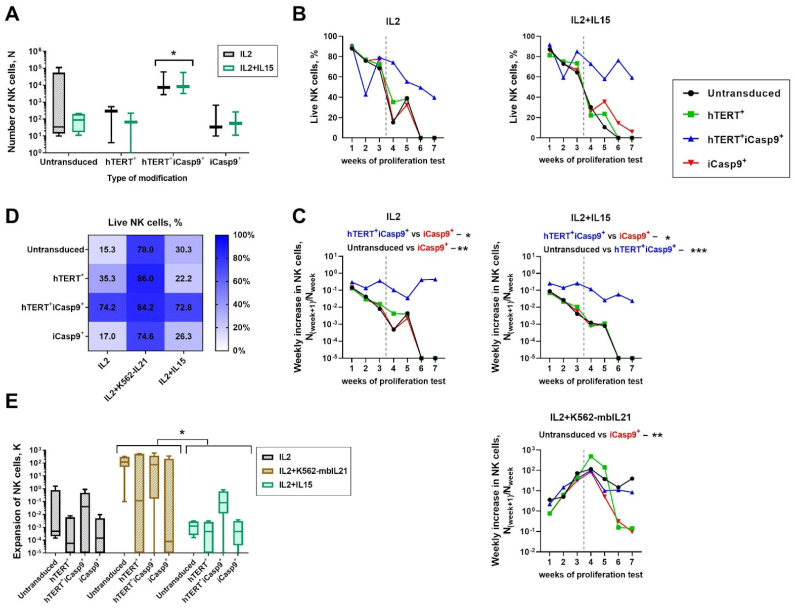
Proliferation assay started 1 month after ex vivo isolation of NK cells modified with *hTERT* and/or *iCasp9* transgenes. Stimulation with IL2, IL2+K562-mbIL21 and IL2+IL15 was performed. (**A**) Numbers of NK cells cultured for one month with IL2+IL15. Mixed effects analysis, Sidak’s multiple comparisons test, *n* = 5, median, *p*-value: * *p* < 0.05. For IL2, IL2+K562-mbIL21 and IL2+IL15 types of stimulations: (**B**) the proportion of live NK cells and (**C**) the weekly increase *n*_(week+1)_/N_week_ are presented. Vertical dashed line stands for a time point of 2 months after isolation. Friedman test with Dunn’s multiple comparisons, *n* = 7, median, *p*-value: * *p* < 0.05; ** *p* < 0.01; *** *p* < 0.001. (**D**) The proportions of live NK cells for each type of stimulation in 2 months of ex vivo isolation. (**E**) The comparison of expansion coefficient between IL2, IL2+K562-mbIL21 and IL2+IL15 stimulations in 2 months of ex vivo isolation. Friedman test with Dunn’s multiple comparisons, median, *n* = 5, *p*-value: * *p* < 0.05; ** *p* < 0.01.

We measured NK cell counts, expansion rate and weekly cell increase obtained from 1 month to 3 months after the ex vivo isolation. By comparing the NK cells cultured under IL2 or IL2+IL15 cytokine support, we defined that NK cells bearing both the *hTERT* and *iCasp9* transgenes display an improved survival rate along with the largest cell numbers remaining after the fourth week of stimulation (Figure 3A,D,E). The hTERT-iCasp9-NK cells showed the greatest proportion (>50%) of live cells in cultures stimulated with IL2+IL15 compared to the other types of modified NK cells (Figure 3B). The hTERT-iCasp9-NK cell cultures maintained proliferation after 4 weeks under all types of stimulation studied, whereas nearly all the NK cells unmodified or transduced with one of the genes collapsed (Figure 3C). A significant improvement in the hTERT-iCasp9-NK cells was observed compared with the iCasp9-NK cells that reflects that hTERT overexpression in iCasp9-NK cells rescues them from the possible toxic effect of the iCasp9 suicide construct. Despite the hTERT-NK cells showing increased survival at the first month of stimulation (Figure 3B), hTERT overexpression did not improve NK cell proliferation during long-lasting cultivation compared to the hTERT-iCasp9-NK cells (Figure 3C). Altogether, a combination of both the *hTERT* and *iCasp9* genes surprisingly improved the maintenance of these gene-modified NK cells. 

The extensive proliferation of modified NK cells is needed to obtain enough cells for therapeutic use; namely, for the treatment of hematological malignancies, more than one million of NK cells per kilogram is needed. Stimulation with IL2+K562-mbIL21 has already shown promising results in the stimulation of ex vivo NK cells. In our research, K562-mbIL21 feeder cells were added monthly to boost the NK cell proliferation. We aimed to figure out if there are any transgene-mediated effects on proliferation, functional activity and acquisition of an exhaustion state. We compared the data obtained 2 months after ex vivo isolation. The highest proportion of the live cells along with the weekly increases in cell numbers and expansion rates were observed for NK cells stimulated with IL2+K562-mbIL21 (Figure 3C–E and Figure S2). Stimulation with feeder cells also upregulated hTERT expression in all the NK cells independently of the transgene (Figure S3). The NK cells cultured without the K562-mbIL21 feeder cells showed reduced viability, which also varied depending on the modification type (Figure 3D). Overall, target recognition on the feeder cells increased the survival and proliferation rate of the NK cells. 

Increased survival with exposure to all types of stimuli used in the study was observed for the hTERT-iCasp9-NK cells (Figure 3D), whereas the iCasp9-NK cells showed a limited lifespan. Their numbers rapidly collapsed after 4 weeks of stimulation (2 months after ex vivo isolation). This corresponds with the 2-month qPCR data, where the levels of pro-apoptotic factors encoded by genes *BAK1*, *BAX* and *DIABLO* were elevated for the iCasp9-NK cells (Figure S4). Similar patterns were observed in the hTERT-NK cells despite the expected pro-survival role of the *hTERT* transgene (Figure 3B,C).

### 3.3. EOMES and T-BET Levels Vary between NK Cells Modified by hTERT and/or iCasp9 Genes

**Figure 4:**
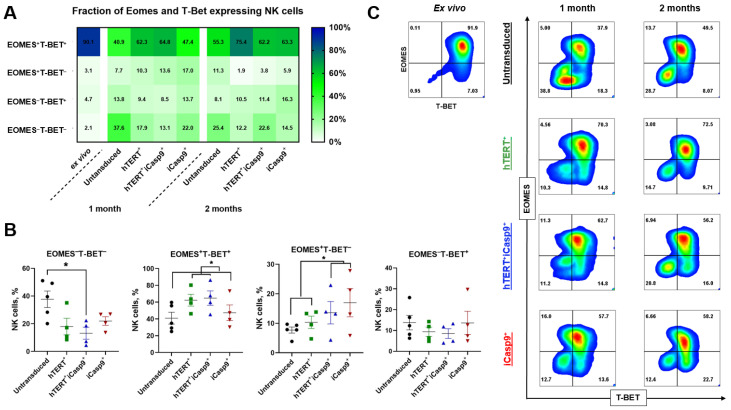
The expression of EOMES and T-BET transcription factors in NK cells modified with *hTERT* and/or *iCasp9* genes. (**A**) NK cells cultivated with IL2+K562-mbIL21 are represented. A change in the proportion of EOMES- and T-BET-expressing cells in 1st and to 2nd month after ex vivo isolation (N_ex vivo_ = 8, N_1 month_ = 5, N_2 months_ = 2, mean). (**B**) The distribution of EOMES^+/−^T-BET^+/−^ subsets of NK cells cultured for 1 month. EOMES^−^T-BET^−^, EOMES^+^ T-BET^+^ (for comparison, data were divided into 2 groups (1st: hTERT^+^ with hTERT^+^iCasp9^+^; 2nd: untransduced with iCasp9^+^)), EOMES^+^ T-BET^−^ (for comparison, data were divided into 2 groups (1st: untransduced with hTERT^+^; 2nd: hTERT^+^iCasp9^+^ with iCasp9^+^)), EOMES^−^ T-BET^+^ *hTERT* and/or *iCasp9* modified NK cells a month after ex vivo isolation (*n* = 5). Unpaired *t*-test, mean, *p*-value: * *p* < 0.05. (**C**) Representative density plots for EOMES and T-BET distributions in NK cells ex vivo, 1 month and 2 months after isolation.

To figure out if there were any contributions of our *hTERT* and *iCasp9* transgenes to the NK cell functional characteristics and degree of their exhaustion on distant time points after ex vivo isolation, we traced the EOMES and T-BET level dynamics by intracellular staining followed by flow cytometry analysis (Figure 4). About 90% of the NK cells after ex vivo isolation were EOMES^+^T-BET^+^, which indicates their good functional and proliferative state (Figure 4A). The exhausted EOMES^−^T-BET^−^ NK cells made up only 2%. EOMES^+^T-BET^−^ NK cells made up 3%. These cells are associated with less differentiated subsets that gradually obtain T-BET expression during maturation. EOMES^+^T-BET^+^ NK cells lose EOMES expression and are finally replaced with terminally differentiated EOMES^−^T-BET^+^ cells (4.7%) (Figure 4A). 

However, the distribution of NK cells within EOMES and T-BET subsets changed a month after the ex vivo isolation and transduction procedure. The proportion of the EOMES^−^T-BET^−^ subset raised from 2% to 37.6% in the untransduced NK cells, to 18% in the hTERT-NK cells, to 13.1% in the hTERT-iCasp9-NK cells and to 22% in the iCasp9-NK cells. The untranduced NK cells demonstrated the highest levels of double negative cells compared to the hTERT-iCasp9-NK cells. The hTERT-modified cells showed a tendency to decrease the proportion of EOMES^−^T-BET^−^ cells compared to the NK cells that were not modified with hTERT (Figure 4B). The TEOMES^+^T-BET^+^ cells were highly represented within the hTERT-modified cells (62.3% in hTERT-NK cells and 64.8% in hTERT-iCasp9-NK cells), but the proportion still decreased by approximately 30% for the hTERT-modified cells and 40–50% for the untransduced and iCasp9-NK cells a month after isolation in comparison with the ex vivo cells (Figure 4B). The fraction of EOMES^+^T-BET^−^ NK cells increased during cultivation. In the NK cells bearing the iCasp9 transgene, it reached 13.6% in the hTERT-iCasp9-NK cells and 17% in the iCasp9-NK cells (Figure 4B). The proportion of EOMES^−^T-BET^+^ did not vary significantly among the transduction groups, and was also slightly increased by 3–8% regarding the ex vivo time point (Figure 4).

Consequently, we noticed the effect of the *iCasp9* transgene had a greater representation in the EOMES^+^T-BET^−^ subsets in the NK cell cultures, while the *hTERT* transgene instead supported the simultaneous expression of both EOMES and T-BET.

### 3.4. NK Cells Maintained the Proportion of EOMES+T-BET+ Subset after 2 Months of In Vitro Cultivation with IL2+K562-mbIL21

Since the highest proliferation rates and increased NK cell survival was observed under IL2+K562-mbIL21 stimulation, we expanded the EOMES and T-BET measurements on NK cells cultivated with IL2+K562-mbIL21. At 2 months after ex vivo isolation, the EOMES^+^T-BET^+^ NK cells constituted the majority in the cell cultures, and their proportions were 55–75%, whereas other EOMES^+/−^T-BET^+/−^ subsets individually represented less than 30% of the cells. Next, we studied the change in the proportion of EOMES^+^ and T-BET^+^ NK cells from the first to the second month after ex vivo isolation. The proportion of T-BET^+^ NK cells increased by 14–30% independently of the transduction type. The fraction of EOMES^+^ cells increased by 13–26% in the untransduced and hTERT-NK cells and remained virtually unchanged (less than 5%) in the iCasp9-modified NK cells. We have also monitored changes in the proportions of the EOMES^+/−^T-BET^+/−^ subsets, which took place in the NK cell cultures between the first and second month after ex vivo isolation. The EOMES^+^T-BET^+^ cell fraction maintained the proportion over time that favorably affected the NK cell activation state, as these cells are supposed to perform functional and proliferative activity without signs of exhaustion occurring, whereas the EOMES^−^T-BET^+^ along with the EOMES^−^T-BET^−^ cell fractions remained unchanged (Figure 4A). Thus, presumably, the hTERT and iCasp9 transgenes governed the balance of EOMES and T-BET expression, but stimulation with K562-mbIL21 feeder cells provided the major supporting effect towards the maintenance of EOMES+T-BET+ fraction.

### 3.5. NK Cells Retained Surface Expression of Activating Receptors Two Months after Ex Vivo Isolation

**Figure 5:**
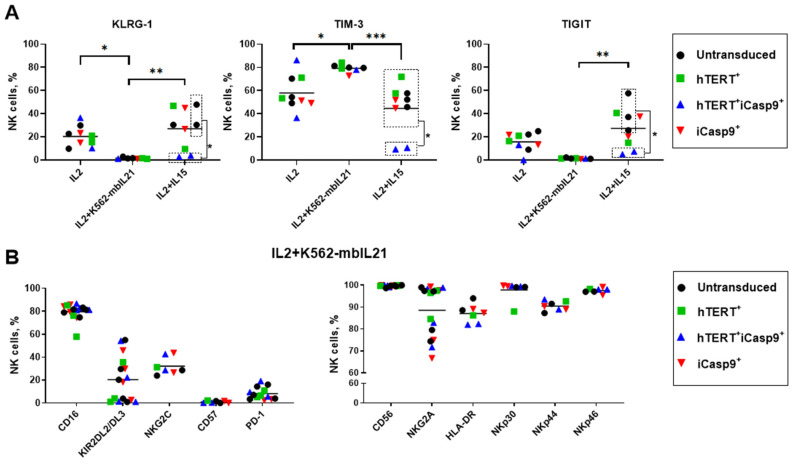
The proportions of NK cells modified with *hTERT* and/or *iCasp9* genes 2 months after ex vivo isolation. Data for IL2, IL2+K562-mbIL21 and IL2+IL15 stimulated NK cells are represented. (**A**) The expression levels of immune checkpoints TIM-3, TIGIT and KLGR-1. Dotted boxes stand to determine the groups compared. Two-way ANOVA with Tukey’s multiple comparisons test, *n* = 3, mean, *p*-value: * *p* < 0.05; ** *p* < 0.01; *** *p* < 0.001. (**B**) Surface expression (means) of CD16, KIR (KIR2DL2/DL3), PD-1, NKG2C, CD57, CD56, NKG2A, HLA-DR, NKp30, NKp44 and NKp46 for NK cells stimulated by IL2+K562-mbIL21.

The transduced NK cells were examined for the presence of surface markers indicating their active functional state. We did not determine any significant transgene-mediated variations in surface markers between NK cells transduced with *hTERT* and/or with i*Casp9* transgenes. Firstly, we compared the expression levels of the immune checkpoint surface receptors PD-1, TIM-3, TIGIT and KLGR-1 that are usually upregulated under cytokine stimulation during chronic diseases. Signals from these immune checkpoints drastically inhibit the proliferative and cytotoxic activity of NK cells [33]. NK cells stimulated with IL2 or IL2+IL15 upregulated TIGIT and KLGR-1 compared to the IL2+K562-mbIL21-stimulated NK cells. Interestingly, hTERT-iCasp9-NK cells were characterized with lower expression levels of these markers in IL2+IL15 stimulation conditions (Figure 5A and Figure S5). The cell fractions bearing PD-1 and TIGIT represented less than 20% of the NK cells stimulated with IL2+K562-mbIL21. However, a great proportion (>50%) of the NK cells expressed the TIM-3 immune checkpoint (Figure 5A and Figure S5). The highest TIM-3 expression was among the IL2+K562-mbIL21 stimulated NK cells compared to IL2 or IL2+IL15 stimulated cells (Figure 5A and Figure S5).

Since the modified NK cells cultured for 2 months with IL2+K562-mbIL21 exhibited the greatest expansion and survival rates that benefit their therapeutic use, we evaluated an additional set of surface markers corresponding with the NK cell activation state (CD16, KIR (KIR2DL2/DL3), NKG2C, CD57, CD56, NKG2A, HLA-DR, NKp30, NKp44 and NKp46) (Figure 5B and Figure S6).

Around 80% of the NK cells after two months of cultivation in the presence of IL2+K562-mbIL21 in all cultures were positive for CD16. All the NK cells had an extremely high density of CD56 marker on their cell surface and showed high expression levels of NKp30, NKp44 and NKp46. Low proportions of CD57^+^ cells were also noted in these cultures. More than 60% of the NK cells expressed the NKG2A receptor, and less than 50% of the NK cells were KIR2DL2/DL3^+^ (Figure 5B).

In this way, we demonstrated that the introduction of transgenes did not negatively affect the expression of a variety of surface markers on the modified cells. All differences in the phenotype detected were mainly mediated by different types of stimulation. Stimulation with the combination IL2+K562-mbIL21 resulted in the accumulation of NK cells with a phenotype that may be considered as being most favorable for immunotherapeutic use.

### 3.6. Despite Similar Expression Levels of EOMES and TBX21 Genes, Two Months after Ex Vivo Isolation, NK Cells Exhibited an Increase in Pro-Apoptotic Factors and a More Suppressive Phenotype

**Figure 6:**
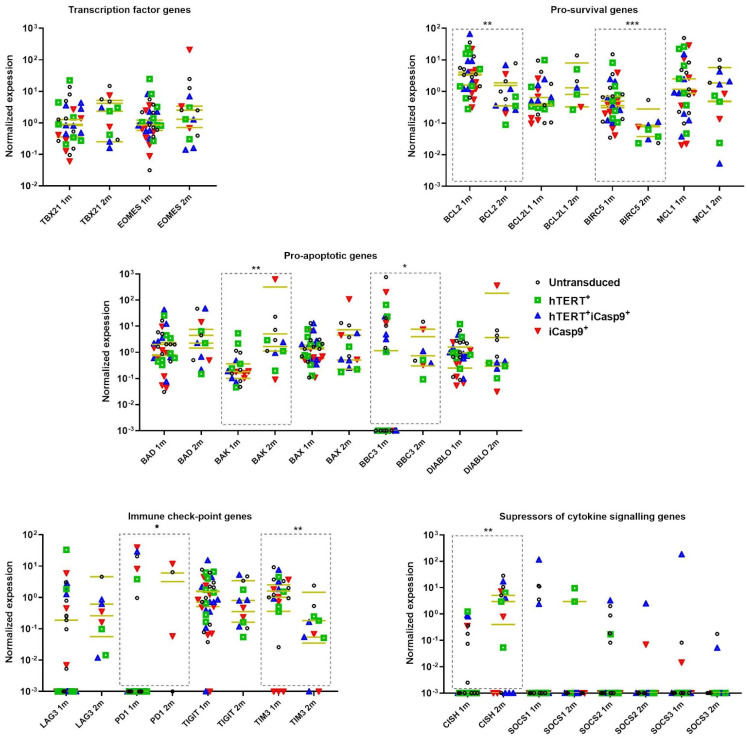
The dynamics of normalized mRNA expression levels of the *EOMES* and *TBX21* genes encoding transcription factors EOMES and T-BET along with expression levels of pro-survival *BCL2*, *MCL1*, *BCL2L1* (BCL-X_L_) and *BIRC5*, pro-apoptotic *BAX*, *BAD*, *BAK*, *DIABLO* and *BBC3* (PUMA), immune-checkpoint-encoding genes *TIM3*, *TIGIT* and *LAG3* and exhaustion-associated *SOCS1-3* and *CISH*. Data obtained at time points of 1 month (1 m) and 2 months (2 m) after ex vivo isolation of hTERT and/or iCasp9 modified NK cells cultured with IL2+K562-mbIL21. Dotted boxes stand to highlight groups compared. Mann–Whitney, N1m = 10, N2m = 3, median, *p*-value: * *p* < 0.05; ** *p* < 0.01; *** *p* < 0.001.

We also studied the expression levels of the *EOMES* and *TBX21* genes encoding the transcription factors EOMES and T-BET along with the expression levels of the pro-survival *BCL2*, *MCL1*, *BCL2L1* (BCL-X_L_) and *BIRC5*, pro-apoptotic *BAX*, *BAD*, *BAK*, *DIABLO* and *BBC3* (PUMA) genes, immune-checkpoint-encoding genes *TIM3*, *TIGIT* and *LAG3* and exhaustion-associated genes *SOCS1-3* and *CISH* (Cis). The NK cells stimulated with IL2+K562-mbIL21 for 1 month displayed decreased mRNA levels of pro-survival factors, namely, BCL-2 and BIRC-5, along with an increase in the pro-apoptotic factors BAK and PUMA. The mRNA levels of the EOMES and T-BET transcription factors remained unaffected. The TIM-3 immune checkpoint level decreased over time, while the *CISH* gene encoding the inhibitor of cytokine signaling Cis was raised. Altogether, the NK cells shifted the live/dead balance towards cell death, but the levels of immune-checkpoint-encoding genes did not increase, and even decreased (Figure 6). We also mentioned the upregulation of EOMES and the pro-apoptotic factors encoded by genes *BAK1*, *BAX* and *DIABLO* in comparison with the hTERT-iCasp9-NK cells, which confirms the observation that hTERT overexpression beneficially affects NK cells bearing the *iCasp9* transgene.

It can be concluded that despite commensurate levels of the expression of *EOMES* and *TBX21* 2 months after cell isolation from peripheral blood, compared with the measurement carried out 4 weeks earlier, there is a shift in the balance of pro-/anti-apoptotic factors towards an increase in pro-apoptotic genes in all types of modified NK cells. The observed increase in *CISH* gene expression, the product of which is known to block the JAK-STAT cascade triggered by cytokine stimulation, may indicate the acquisition of exhaustion in NK cells. The upregulation of *CISH* gene expression is most likely associated with cell cultured in a medium supplemented with IL2, which can cause its increase.

### 3.7. The Functional Activity of hTERT- and/or iCasp9-Transduced NK Cells Is Donor-Mediated

**Figure 7:**
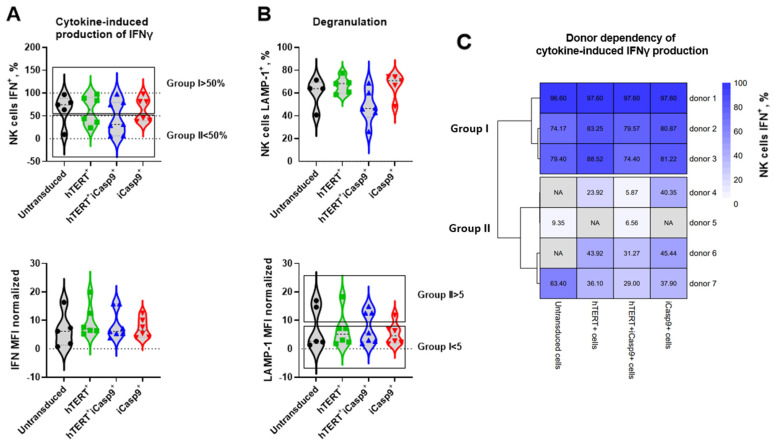
The functional activity of NK cells modified with *hTERT* and/or *iCasp9* genes a month after their ex vivo isolation. (**A**) The proportion of NK cells accumulating IFNγ in response to IL2, IL12 and IL18 cytokines (Top). The intensity of cytokine-dependent IFNγ production per cell measured by mean fluorescence intensity normalized to FMO control (bottom). (**B**) The fraction of NK cells capable of degranulation measured by LAMP-1 surface exposure in response to the recognition of K562 target cells (Top). The intensity of degranulation in response to K562 cells production per cell is measured by mean fluorescence intensity normalized to basal degranulation (bottom). The cells were also divided into groups I (IFNγ > 50% and MFI LAMP-1 < 5) and II (IFNγ < 50% and MFI LAMP-1 > 5). (**C**) The donor dependency of IFNγ production. Ordinary one-way ANOVA, *n* = 7 donors.

Therapeutic cells need to be functionally active at the moment of their infusion into patients. So, we compared the functional activity of NK cells transduced with the *hTERT* and/or *iCasp9* genes. The NK cells underwent a cytokine-mediated IFNγ assay (Figure 7A and Figure S7A) and degranulation test aimed at the K562 cell line a month after ex vivo isolation (Figure 7B and Figure S7B). No significant differences were detected between the NK cells modified with the *hTERT* and/or *iCasp9* transgenes, although a slightly decreased fraction of LAMP1^+^ cells was observed in the hTERT-iCasp9-NK cells compared to the other NK cell cultures: 60% in the untransduced NK cells, 67% in the hTERT-NK cells, 48.4% in the hTERT-iCasp9-NK cells and 66.9% in the iCasp9-NK cells (Figure 7B). We have also noticed that the proportion of IFNγ^+^ cells can be subdivided almost evenly into two groups within the untransduced cells and each type of the modified NK cells (Group I: IFNγ > 50% and Group II: IFNγ < 50%) (Figure 7). Similar groups were observed for the degranulation data, where Group I corresponded to poorly degranulated cells with MFI < 5, whereas Group II was characterized by MFI > 5. Thus, in Group I, most of the NK cells were capable of intensive IFNγ production and exhibited low degranulation per cell. In Group II, on the contrary, the NK cells were less committed to IFNγ production, but capable of intensive degranulation.

Such grouping allowed us to figure out predictive factors that could mediate the strength of the functional response in the studied NK cell types. Interestingly, the 1-month functional activity was altered between donors (Figure 7C).

**Figure 8:**
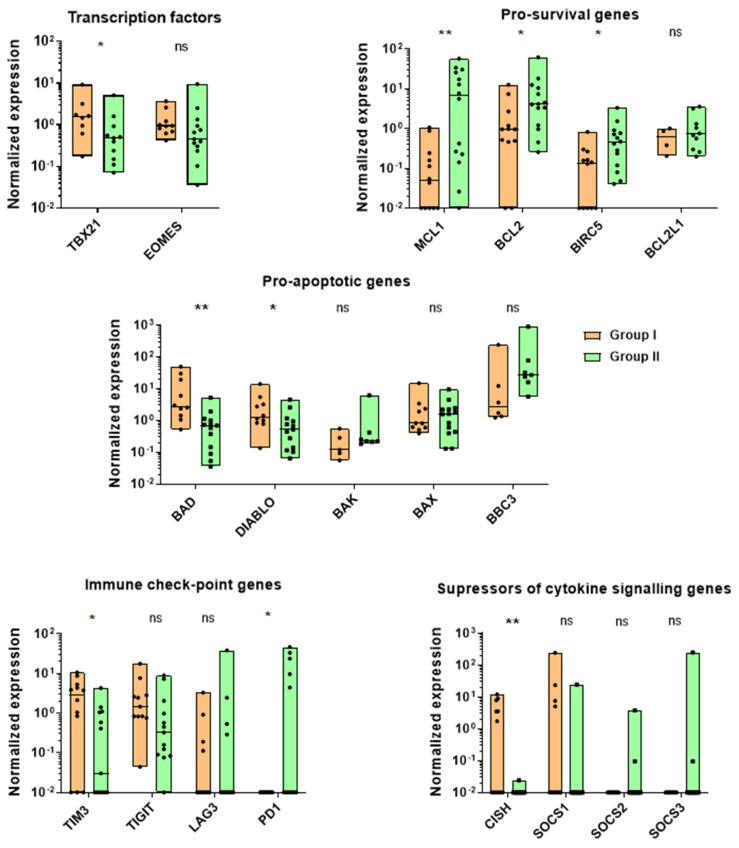
Normalized mRNA expression levels of the *EOMES* and *TBX21* genes encoding transcription factors EOMES and T-BET along with expression levels of pro-survival *BCL2*, *MCL1*, *BCL2L1* (BCL-X_L_) and *BIRC5*, pro-apoptotic *BAX*, *BAD*, *BAK*, *DIABLO* and *BBC3* (PUMA), immune-checkpoint-encoding genes *TIM3*, *TIGIT* and *LAG3* and inhibitors of cytokine signaling *SOCS1-3* and *CISH* are presented for Group I (IFNγ > 50% and MFI LAMP-1 < 5) and Group II (IFNγ < 50% and MFI LAMP-1 > 5) NK cells a month after ex vivo isolation. Mann–Whitney, *n* = 7 (Group I: *n* = 13, Group II: *n* = 14), median, *p*-value: * *p* < 0.05; ** *p* < 0.01.

The expression levels of the EOMES and T-BET transcription factors, known for their direct impact on the NK functional state, were investigated [8]. The gene expression was determined via qPCR a month after ex vivo isolation. The *TBX21 (encodes T-BET)* mRNA expression level was significantly higher in Group I compared to Group II, whereas no evident difference was determined for EOMES (Figure 8). 

Along with the EOMES and T-BET expression levels, the mRNA levels of the pro-survival *BCL2*, *MCL1*, *BCL2L1* (BCL-X_L_) and *BIRC5*, pro-apoptotic *BAX*, *BAD*, *BAK*, *DIABLO* and *BBC3* (PUMA), exhaustion-associated *SOCS1-3* and *CISH*, and immune-checkpoint-encoding genes *TIM3*, *TIGIT* and *LAG3* were evaluated (Figure 8). Group I NK cells were characterized by higher expression levels of T-BET, pro-apoptotic *DIABLO*, *BAD*, immune checkpoint *TIM3* and exhaustion-associated *CISH*, and lowered levels of the pro-survival genes *MCL1*, *BCL2*, *BIRC5* compared to Group II. For the other genes studied, no significant differences were observed.

As a result, IFNγ production was shown to be unregulated simultaneously with T-BET levels, and varied among donors (Table S2). However, we did not manage to validate any associations between the IFNγ NK cell responses and donor characteristics such as gender, age, seropositivity to viral infections (cytomegalovirus (CMV), SARS-CoV-2 and Epstein–Barr virus (EBV)).

## 4. Discussion

Immune cell exhaustion and low proliferative capacity in the weeks after isolation are still the key sticking points of NK cell therapeutic approaches. Additional manipulations such as retroviral genetic modifications and expansion-stimulating procedures performed on NK cells may decrease the life expectancy and activation state because of the acquisition of exhaustion in therapeutic NK cells. For example, the successful treatment of patients with malignancies requires the infusion of huge numbers of modified cells (ranging from 1 × 10^6^/kg to 9.3 × 10^6^/kg) [34]. It is complicated to obtain such abundant NK cell numbers because of the limited NK cell content in peripheral blood and the comparatively low proliferative potential of donor-derived NK cells. The acquisition of a pure fraction of modified NK cells is another procedure complicated by the innate ability of NK cells to resist viral modifications [35], so the fractions of successfully transduced NK cells is low compared to T cells widely applied in therapeutic approaches [36]. Moreover, it takes around a month of in vitro NK cell culturing for the appropriate stimulation and conduction of the transduction procedure [34]. However, the sustained cytokine stimulation of NK cells may lead to their exhaustion. In order to overcome such limitations and restore the proliferative potential and lower the acquisition of exhaustion in NK cell cultures, we transduced NK cells with the *hTERT* gene encoding the catalytic subunit of telomerase [22,23]. However, the therapeutic usage of such immortal hTERT-NK cells brings some safety concerns, so a specific control of hTERT-NK cells is needed within the patient. The iCasp9 switch construct based on caspase 9 has already shown promising results in clinics [37]. Therefore, we combined two approaches: proliferative and functional enhancement of NK cells by hTERT overexpression and safety control of NK cells by their modification with the *iCasp9* gene.

We studied the effects of the *hTERT* and *iCasp9* transgenes on NK cell proliferation and expansion a month after ex vivo isolation (time point when therapeutic NK cells are normally ready to use). We cultivated NK cells with three types of stimuli: IL2, IL2 with feeder K562-mbIL21 cells added monthly, and IL2 in combination with IL15. Such conditions allow us to predict how the transduced NK cells may act without target recognition (IL2 stimulation), upon target recognition (IL2+K562-mbIL21) and under the action of the additional stimulus IL15 (IL2+IL15), known for its role in NK cell survival and wide representation within inflammation sites. 

We compared the effects of IL2 and a combination of IL2 with IL15 on the NK cell proliferation a month after ex vivo isolation. Due to the multiple positive effects IL15, it is considered an essential supplement to NK cell therapy. IL15 improves longevity, proliferation potential and antitumor function [38]. IL15 signaling results in the upregulation of STAT5 target genes including various pro-survival factors, namely Mcl-1, the loss of which triggers apoptosis in NK cells [38]. However, our data showed that NK cells, both unmodified and modified with one of the transgenes (*hTERT* or *iCasp9*), are more likely to collapse in a medium containing IL15 along with IL2 (Figure 3B,C). Interestingly, only the hTERT-iCasp9-NK cells survived over a month of culturing and maintained proliferation in both IL2- and IL2+IL15-stimulating conditions in comparison with the iCasp9-NK cells. The results suggest that iCasp9 spontaneous dimerization promotes the selection of NK cells with an apoptotic balance shifted towards NK cell survival, while hTERT overexpression in these cells can provide essential rescue for iCasp9 cells via hTERT non-canonical functions [24,39,40,41,42], as was similarly described for Bcl-2 transgenic mice under IL15 deprivation [38]. At the same time, a higher hTERT level in the hTERT-NK cells compared to the hTERT-iCasp9-NK cells seems to be unable to improve survival to such an extent as it was shown for the hTERT-iCasp9-NK cells, so the impact of the *iCasp9* transgene is apparently essential for NK cell survival. It was reported earlier [8] that the responsiveness of NK cells to IL15 is complicated and greatly depends on a proper balance between EOMES and T-BET expression. Both EOMES- and T-BET-deficient NK cells are characterized by a decrease in viability under IL15 stimulation. The other explanation for NK cell contraction after 2 months of cultivation could be caused by Cis encoded by *CISH* gene upregulation that dampens IL15 responses by binding to the IL15R and inhibition of JAK1 kinase [11,38]. We have previously mentioned that hTERT-iCasp9-NK cells combine features of both hTERT-NK cells and iCasp9-NK cells (Figure 4B) suggesting the fine tuning of EOMES and T-BET functions in terms of the regulation of NK cell survival. The expression of cytokine receptors (IL15 and IL2) and regulation of the acquisition of exhaustion may mediate the increased survival of hTERT-iCasp9-NK cells. Interestingly, the reduced lifespan of the iCasp9-NK cell cultures was also associated with the smallest *hTERT* expression level (Figure 2A), possibly because of the toxicity caused by the spontaneous dimerization of the iCasp9 suicide construct [31].

The highest proportion of live cells along with a high expansion rate was observed in the transduced NK cells stimulated by IL2+K562-mbIL21 (Figure 3). However, the populations of the untransduced and iCasp9-NK cells rapidly contracted 3 months after ex vivo isolation (Figure 3B,C). To study the conditions mediating the low response of NK cell cultures to the third stimulation with K562-mbIL21, we examined the ratio of EOMES^+/−^ and T-BET^+/−^ subpopulations 2 months after ex vivo isolation. As shown in Figure 4A, the EOMES^+^T-BET^+^ NK cells were abundantly represented in all the populations studied. Moreover, an increase in Cis levels emerged under sustained STAT5 activation induced by IL2 or IL15 [11,38]. Thus, initially, T-BET may promote the expression of receptors to cytokines (IL2, IL21, IL12, IL15, IL18) and consequently boost signal transmission during cytokine stimulation and activate a negative feedback loop increasing Cis and Bak levels and decreasing Bcl-2 and BIRC5 levels (Figure 6). As Cis suppresses Jak/STAT5 signaling responsible for NK cell survival, the raised DIABLO and Bad levels observed may be partially explained by Cis upregulation [11]. These facts correspond with our observations of the limited life expectancy of NK cells after 2 months of culturing under the permanent presence of IL2.

We studied the proportion of NK cells modified with the *hTERT* and/or *iCasp9* genes that expressed the EOMES and T-BET transcription factors. The proportion of active functional and proliferative EOMES^+^T-BET^+^ NK cells that were dominant after ex vivo isolation decreased to >30% after a month of cultivation in vitro (Figure 4A).

The hTERT-modified NK cells showed a higher percentage of EOMES^+^T-BET^+^ cells (Figure 4B), which corresponds with the suggestion that hTERT overexpression positively affects the NK cell functional state [22]. EOMES, according to the literature, is associated with hTERT via the b-catenin/Wnt pathway, improving the survival, maturation, and cytotoxicity of NK cells [43]. T-BET is involved in the regulation of the cell cycle through mTORC [44,45].

The introduction of the *iCasp9* gene into the NK cells also increased the fractions of the EOMES^+^T-BET^−^ cells (Figure 4B). Possibly, the spontaneous dimerization of iCasp9 [31] provided a selection of less apoptotic cells that corresponded with qPCR data from Group I (IFNγ^+^ NK cells > 50% and LAMP1 MFI < 5) and Group II (IFNγ^+^ NK cells < 50% and LAMP1 MFI > 5) cells (Figure 8). NK cells with increased T-BET expression were characterized by higher levels of pro-apoptotic factors such as DIABLO and Bad, whereas lower T-BET expression was linked to higher expression levels of the pro-survival factors Bcl-2, Mcl-1 and BIRC-5. These data show that since the iCasp9 gene can spontaneously dimerize and trigger apoptosis, the iCasp9^+^ and hTERT^+^iCasp9^+^ cells need to maintain EOMES levels to survive as this transcription factor induces the expression of many genes providing survival signals (such as some NK cell receptors) [8,43].

Generally, an increase in EOMES expression was characteristic for iCasp9-modified NK cells (iCasp9^+^ and hTERT^+^iCasp9^+^), and an increase in double positive (EOMES^+^T-BET^+^) cells was observed for the hTERT-modified NK cells (hTERT^+^ and hTERT^+^iCasp9^+^). Thus, the *iCasp9* gene is characterized by the preservation of EOMES^+^T-BET^−^ cells, and *hTERT* maintains double positive cells. The hTERT-iCasp9-NK cells seem to occupy an intermediate place between hTERT-NK cells and iCasp9-NK cells, along with the significantly lesser proportion of exhausted EOMES^−^T-BET^−^ NK cells compared with the untransduced NK cells. We also mentioned some inconsistencies between qPCR expression analysis and FACS data reflecting the intracellular presence of proteins that may be caused by different kinetics of transcription, translation and degradation processes.

We determined the surface expression of several activation markers of NK cells and exhaustion-associated markers after 2 months of cultivation with IL2, IL2+K562-mbIL21 and IL2+IL15 (Figure 5). The IL2- and IL2+IL15-stimulated NK cells exhibited increased levels of KLRG-1 and TIGIT associated with a state of exhaustion and corresponded with low proliferative and survival rates.

The NK cells stimulated with IL2+K562-mbIL21 for 2 months were characterized by the low (<20%) proportion of PD-1^+^ and TIGIT^+^ NK cells and high (>80%) percentage of TIM-3^+^ cells, but no terminally differentiated KLRG-1^+^ cells were detected [1]. Since it is known that a high expression of KLRG-1 correlates with a low proliferative capacity, the impaired secretion of IFNγ and increased apoptosis in NK cells [4,46], the absence of this marker on the cell surface characterizes NK cells as unexhausted. We also observed the higher expression of TIM-3. The dual role of TIM-3 has recently been shown: TIM-3 is broadly expressed on the surface of functionally active NK cells, but its binding to a cognate ligand impairs cytotoxicity [47]. Altogether, the modified NK cells showed an unexhausted phenotype in comparison to that described in Alvarez M.’s review [1]. However, the overall proportion of T-BET+ cells displayed a tendency to rise from 1 month up to 2 months of culturing according to flow cytometry data (Figure 3A). This also corresponds with another research group that showed that terminal differentiation is facilitated by T-BET and instead is prevented by EOMES [8].

Similarly to the research of Huntington, N. et al. [7], among the IL2+K562-mbIL21 cells stimulated for 2 months, the NK cells were detected at high proportions (around 80%) of the CD16^+^ cells. The CD16 receptor responsible for antibody-dependent cell-mediated cytotoxicity is regulated by both transcription factors (EOMES and T-BET), whereas EOMES stands as a stronger driver of CD16 expression than T-BET. Recently, it was shown that deficiency of one of T-BET or EOMES impairs NK cell functional activity; it can be rescued by the upregulation of another one while double positive (EOMES+T-BET+) NK cells perform an entire spectrum of functionalities. In our case, the NK cell cultures did not impair functional activity such as degranulation, IFNγ production and cytotoxicity [7,22,48]. Correspondingly with the data obtained from Group 2 cells, a reduced T-BET level along with normal EOMES expression makes NK cells less susceptible to apoptosis induction. The decreased expression levels of the *TIM3*, *TIGIT*, *SOCS1* and *CISH* genes were observed for the NK cells with reduced T-BET levels. However, lowered T-BET levels may dampen the IL12 response and consequently reduce IFNγ production in NK cells [48]. Most of the cells (over 80%) were bearing a set of natural cytotoxicity receptors, NKp44, NKp46, NKp30 and HLA-DR marker, which are normally exposed on activated NK cells and facilitated by increased levels of both transcription factors EOMES and T-BET, which coincides with the prediction that 2-month IL2+K562-mbIL21 stimulated NK cells did not display exhaustion [7]. We also determined the high density of the CD56 marker. A similar phenotype was described earlier for hTERT-NK cells stimulated with K562-mb15-41BBL [22]. The described phenotype likely corresponds with the distribution of EOMES^+/−^ T-BET^+/−^ NK cells, with the EOMES^+^T-BET^+^ cells being the most widely represented [7].

## 5. Conclusions

Altogether, the NK cells modified with the *hTERT* and/or *iCasp9* genes were monitored over 2 months of stimulation. The ratio of the EOMES^+/−^ and T-BET^+/−^ subsets shifted right after ex vivo isolation, with an overall 30% decrease in the EOMES^+^T-BET^+^ cells 1 month after isolation, but then was maintained for up to 2 months for NK cells stimulated with IL2 and K562-mbIL21 feeder cells added monthly. These NK cells were also characterized by a less exhausted phenotype compared to the IL2- or IL2+IL15-stimulated NK cells. Interestingly, the modified hTERT-iCasp9-NK cells showed significantly enhanced proliferation rates compared to the iCasp9-NK cells and a tendency for elevated longevity compared to NK cells transduced with just one of the transgenes. Such an effect could be achieved by the simultaneous action of both transgenes, where hTERT promotes survival by its canonical and non-canonical functions and iCasp9 provides a selection of the most “live” NK cells via spontaneous dimerization. However, the precise action of the iCasp9 construct on NK cell survival still requires investigation. Thus, hTERT provides some sort of rescue for iCasp9-modified NK cells which manifests during prolonged cultivation. The functional activity did not vary significantly depending on the type of transgene, but rather depended on the T-BET expression level in each donor at the time point of 1 month of cultivation. Thus, the transduction of a combination of the *hTERT* and *iCasp9* transgenes might be a promising approach for obtaining gene-engineered NK cells for anticancer therapy, and the EOMES and T-BET expression levels can be used as predictable markers for choosing the most efficient methods of NK cell culture and activation.

## Data Availability

The data supporting the conclusions of this article will be made available by the authors on request.

## Supplementary Materials

The following supporting information can be downloaded at https://www.mdpi.com/article/10.3390/biomedicines12030650/s1. Table S1. List of used primers. Figure S1. Representative plots for apoptosis detection. NK cells untransduced and transduced with *hTERT* and/or *iCasp9* genes were stained with Annexin V and SYTOX after 24 h incubation with chemical inductor of dimerization. The lowest left square represents the fraction of live NK cells. Figure S2: Proliferation assay started 1 month after ex vivo isolation for NK cells modified with *hTERT* and/or *iCasp9* transgenes. Stimulation with (A) IL2, (B) IL2+K562-mbIL21 and (C) IL2+IL15 was performed. For each stimulation type, the proportion of live NK cells (left panel) and the expansion coefficient (right panel) are presented. Friedman test with Dunn’s multiple comparison, *n* = 7, median, *p*-value: * *p* < 0.05; ** *p* < 0.01; *** *p* < 0.001. Figure S3. The levels of the catalytic subunit of telomerase, hTERT, measured by intracellular staining and subsequent FACS analysis in unmodified and hTERT- and/or iCasp9-transduced NK cells cultured with IL2+K562-mbIL21 feeder cells for 2 months. Blue histogram represents second antibody control. Red histogram represents hTERT staining. Figure S4. The dynamics of normalized mRNA expression levels of the *EOMES* and *TBX21* genes encoding transcription factors EOMES and T-BET along with expression levels of pro-survival *BCL2*, *MCL1*, *BCL2L1* (BCL-X_L_) and *BIRC5* genes, pro-apoptotic *BAX*, *BAD*, *BAK1*, *DIABLO* and *BBC3* (PUMA) genes, immune-checkpoint-encoding genes *TIM3*, *TIGIT* and *LAG3* and exhaustion-associated *SOCS1-3* and *CISH*. Data obtained at time points of 1 month (1 m) and 2 months (2 m) after ex vivo isolation of hTERT- and/or iCasp9-modified NK cells cultured with IL2+K562-mbIL21. Two-way ANOVA, Tukey’s multiple comparisons, N1m = 10, N2m = 3, median, *p*-value: * *p* < 0.05; ** *p* < 0.01; *** *p* < 0.001. Figure S5. Representative plots describing gating strategy and representative fractions of TIM-3^+^, TIGIT^+^, KLRG-1^+^ and PD-1^+^ NK cells unmodified and modified with *hTERT* and/or *iCasp9* genes at a time point of 2 months after isolation. NK cells were cultured with 3 types of stimuli: IL2, IL2+K562-mbIL21, IL2+IL15. Figure S6. Representative plots describing gating strategy for NK cells, single cells and live cells and representative fractions for stained surface markers CD56, CD57, activating receptors CD16, HLA-DR, NKG2C, NKp30, NKp44, NKp46 and inhibitory receptors KIR2DL2/DL3 and NKG2A. Data presented for NK cells unmodified and modified with *hTERT* and/or *iCasp9* genes at a time point of 2 months after isolation. NK cells were cultured with IL2+K562-mbIL21. Figure S7. Representative plots describing gating strategy for NK cell functional assays. (A) Cytokine (IL2, IL12, IL18)-induced IFNγ production. NK cells were divided into GFP^−^ and GFP^+^ cells and studied for IFNγ production. Light blue represented FMO control. Orange histogram represents NK cells stained with anti-INFγ antibodies. (B) NK cell degranulation determined by LAMP-1 surface exposure in response to target cell (K562 cells) recognition. Blue corresponds with control NK cells incubated without K562 cells and red represents NK cells mixed with targets. Table S2. Characteristics of the donors whose cells were used in the study. Seven healthy volunteers were randomly selected. They had no symptoms of colds or malaise before blood sampling. The age of the donors ranged from 29 to 53 years. Donors were subdivided into 2 groups: Group I (IFNγ > 50% and MFI LAMP-1 < 5) and Group II (IFNγ < 50% and MFI LAMP-1 > 5). The blood serum was analyzed for the presence of specific IgG to common viruses such as CMV, SARS-CoV-2 and EBV.
