# Supplementary material for: The hTERT and iCasp9 Transgenes Affect EOMES and T-BET Levels in NK Cells and the Introduction of Both Genes Improves NK Cell Proliferation in Response to IL2 and IL15 Stimulation
Source: Biomedicines. 2024 Mar 14;12(3):650. doi: 10.3390/biomedicines12030650 (PMC10968005; doi:10.3390/biomedicines12030650)
Supplement: Supplementary file 1 [file biomedicines-12-00650-s001.zip › biomedicines-2863435-supplementary.pdf]

biomedicines

MDPI

Table S1. List of used primers.

Gene	Primer	Primer sequence
ACTB	B-actin F	CAC CAC ACC TTC TAC AAT GAG
	B-actin R	GTC TCA AAC ATG ATC TGG GTC
BAD	Bad F	TGT GGA CTC CTT TAA GAA GGG AC
	Bad R	CAC CAG GAC TGG AAG ACT CG
BAK1	Bak F	GAT CCC GGC AGG CTG ATC C
	Bak R	GTA GCT GCG GAA AAC CTC CT
BAX	Bax F	TCA TGG GCT GGA CAT TGG AC
	Bax R	GCG TCC CAA AGT AGG AGA GG
BCL2	Bcl-2 F	GTG GAG GAG CTC TTC AGG GAC
	Bcl-2-R	ATG ACC CCA CCG AAC TCA AAG
BCL2L1	Bcl-X_L F	AGG CGG ATT TGA ATC TCT TTC TCT
	Bcl-X_L R	GGG CTC AAC CAG TCC ATT GT
BIRC5	BIRC-5 F	CCA CTG AGA ACG AGC CAG AC
	BIRC-5 R	GTT CCT CTA TGG GGT CGT CAT
DIABLO	Diablo F	AGT AAC CCT GTG TGC GGT TC
	Diablo R	ACT GCT CTC CTC ATC AAT GCT
EOMES	EOMES F	AGG CGC AAA TAA CAA CAA CAC C
	EOMES R	ATT CAA GTC CTC CAC GCC ATC
iCASP9	iCasp-HA F	TGG GCT CAC TCT GAA GAC CT
	iCasp-HA R	AGT GCG TAG TCT GGT ACG TC
MCL1	Mcl-1 F	TAC TTT GGC TTG GTT TCC ATG A
	Mcl-1 R	ATT ACT GAG CCT TCC GTC AAG T
PMAIP1	Noxa F	ACG AGG AAC AAG TGC AAG TAG C
	Noxa R	CAG GTT CCT GAG CAG AAG AGT T
BBC3	Puma F	GAT GAA ATT TGG CAT GGG GTC T
	Puma R	AGG GCT GAG GAC CAC AAA TCT
TBX21	T-BET F	CTG GAT GCG CCA GGA AGT TT
	T-BET R	CTG GAG CAC AAT CAT CTG GGT
hTERT	hTERT F	GCC TGA GCT GTA CTT TGT CAA
	hTERT R	GGT TTG ATG ATG CTG GCG AT
TIM3	TIM3-F	TCC AAG GAT GCT TAC CAC CAG
	TIM3-R	AAT GTG GAT ATT TGT GTT AGA TT
TIGIT	TIGIT-F	GGT CCT AGA AAG CTC AGT GGC
	TIGIT-R	TTC TAG TCA ACG CGA CCA CC
PD1	PD1-F	GTT TCC CTT CCG CTC ACC T
	PD1-R	GTC TAA GAA CCA TCC TGG CCG
LAG3	LAG3-F	GGC AAT CAT CAC AGT GAC TCC
	LAG3-R	GAG CTC CAC ACA AAG CGT TC
CISH	CIS-F	TTC AGG GAC CTC GTC CTT TG
	CIS-R	ATG ACT GGC TTG GGC AGT TC
SOCS1	SOCS1-F	TCT CAC CTC TTG AGG GGG TC
	SOCS1-R	AGA GGT AGG AGG TGC GAG TT
SOCS2	SOCS2-F	CTT GAG CCC TCC GGG AAT G
	SOCS2-R	TCC CCA GTA CCA TCC TGT CT
SOCS3	SOCS3-F	ACT GAG CCG ACC TCT CTC CT
	SOCS3-R	GGC AGC TGG GTC ACT TTC TC

Table S2. Characteristics of the donors whose cells were used in the study. 7 healthy volunteers were randomly selected. They had no symptoms of colds or malaise before blood sampling. The age of the donors ranged from 29 to 53 years. The blood serum was analyzed for the presence of specific IgG to such common viruses as CMV, SARS-CoV-2, and EBV.

	Donor	Gender	Age	CMV	SARS-Cov-2	EBV
**Group I**	**1**	**Male**	**33**	**-**	**+**	**+/-**

	**2**	**Female**	**53**	+	+/-	+
	**3**	**Male**	**35**	+	+	+
Group II	4	Male	29	?	?	?
	5	Female	32	+	-	?
	6	Male	40	+	+	+
	7	Female	33	+	-	?

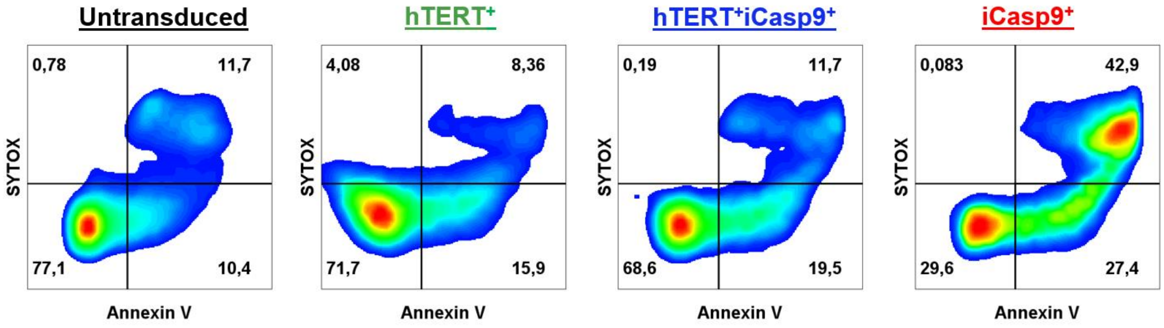

Figure S1. Representative plots for apoptosis detection. NK cells untransduced and transduced with hTERT and/or iCasp9 genes were stained with Annexin V and SYTOX after 24 h incubation with chemical inductor of dimerization. The lowest left square represents the fraction of live NK cells.

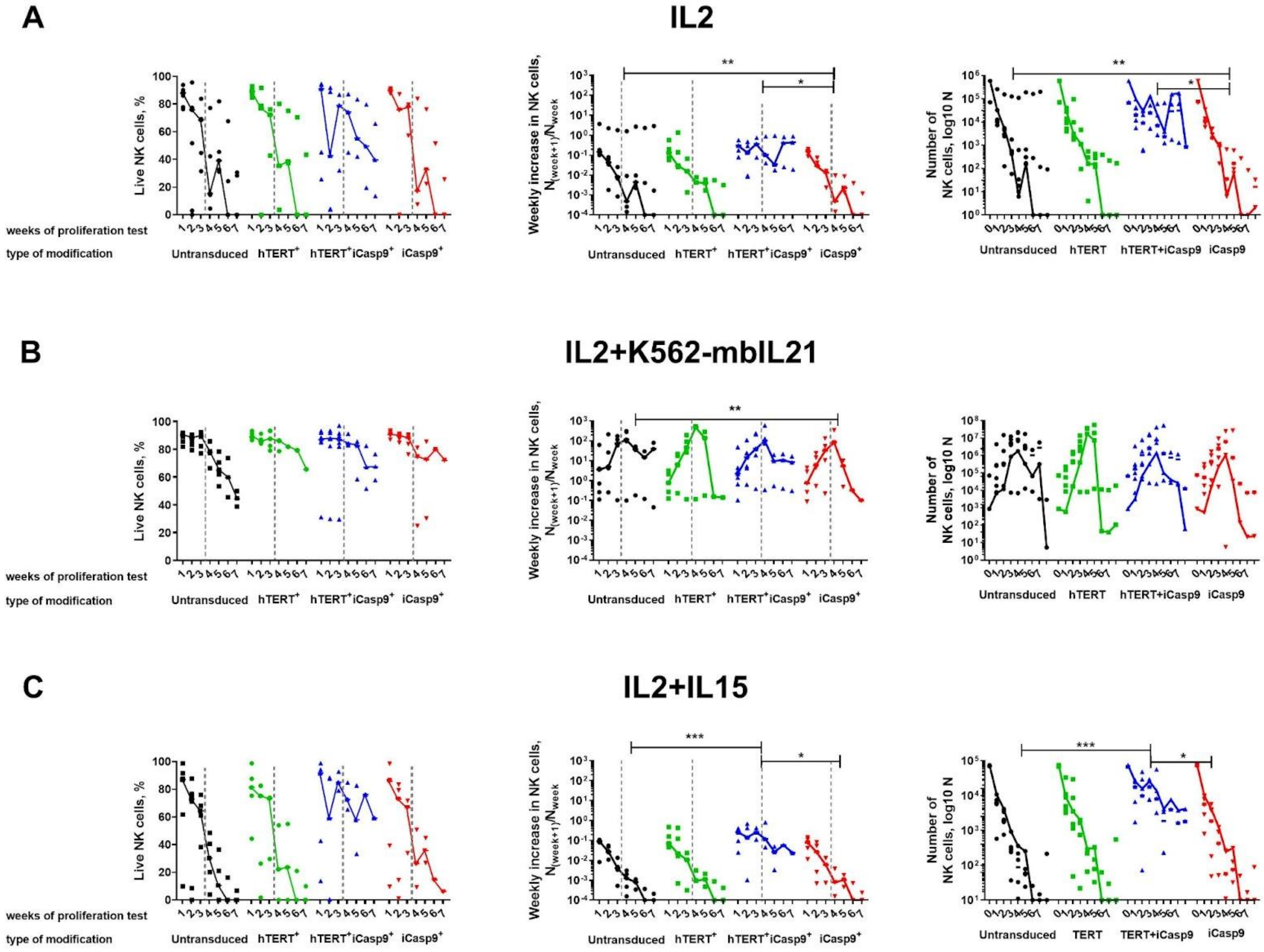

Figure S2. Proliferation assay started in a 1 month after ex vivo isolation for NK cells modified with hTERT and/or iCasp9 transgenes. Stimulation with A) IL2, B) IL2+K562-mbIL21 and C) IL2+IL15 was performed. For each stimulation type the proportion of live NK cells (left panel) and the expansion coefficient (right panel) are presented. Friedman test with Dunn's multiple comparison, n=7, median, p-value: * p < 0.05; ** p < 0.01; *** p< 0.001.

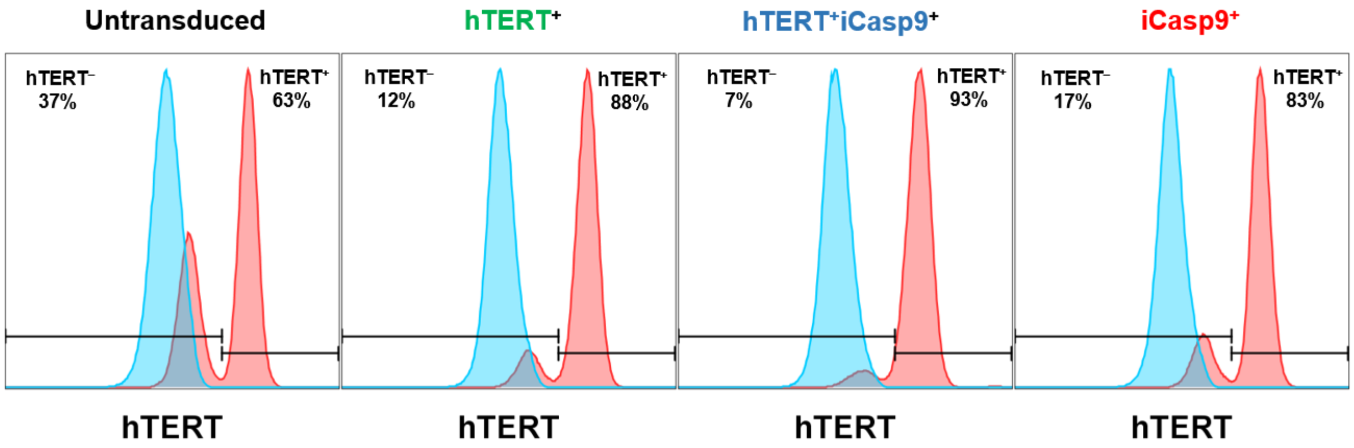

Figure S3. The levels of catalytic subunit of telomerase hTERT measured by intracellular staining and subsequent FACS analysis in unmodified and *hTERT* and/or *iCasp9* transduced NK cells cultured with IL2+K562-mbIL21 feeder cells for 2 months. Blue histogram stands for second antibody control. Red histogram represents hTERT staining.

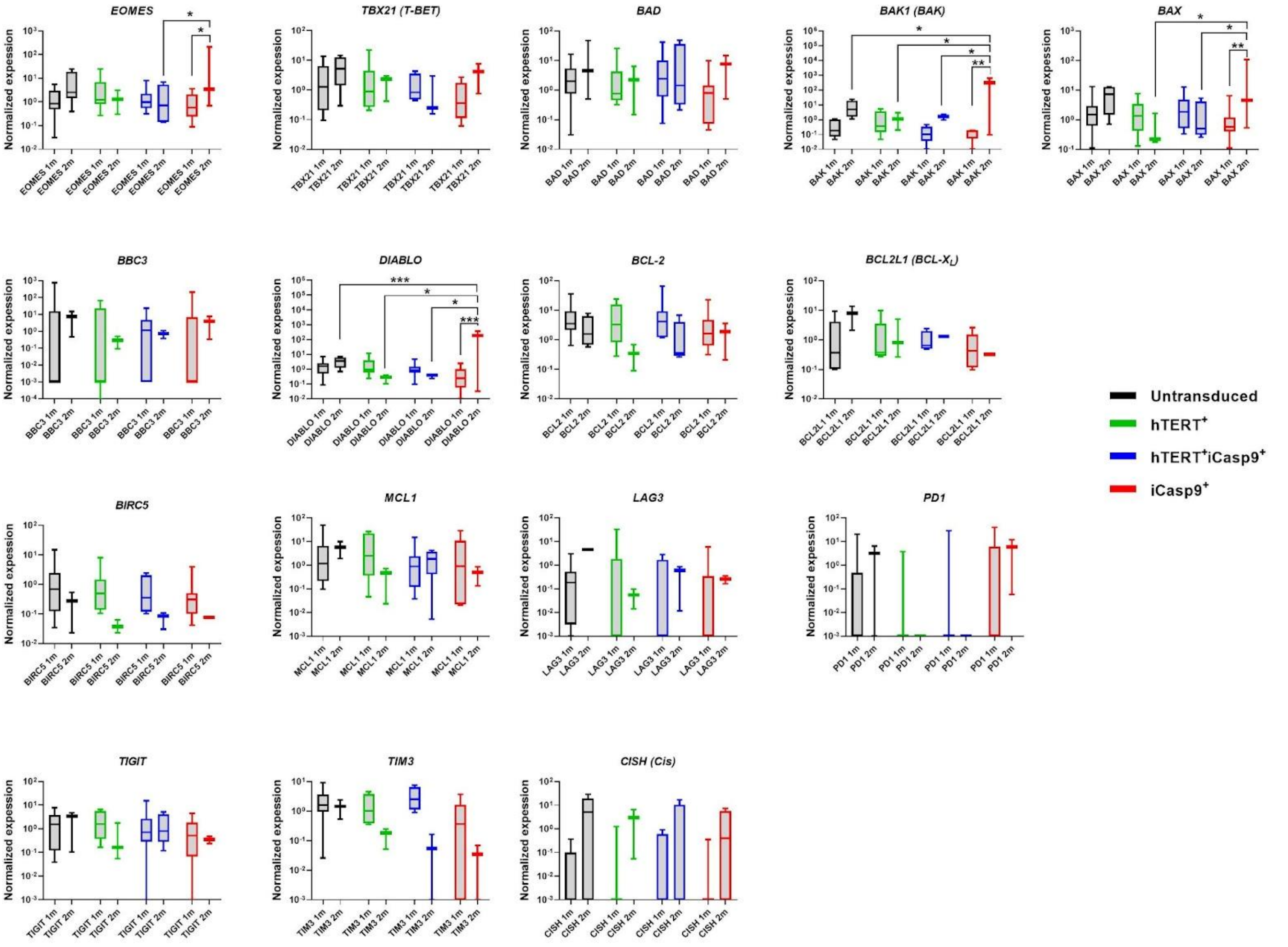

Figure S4. The dynamics of normalized mRNA expression levels of the EOMES and TBX21 genes encoding transcription factors EOMES and T-BET along with expression levels of prosurvival BCL2, MCL1, BCL2L1 (BCL-XL) and BIRC5, pro-apoptotic BAX, BAD, BAK, DIABLO and BBC3 (PUMA), immune check-point encoding genes TIM3, TIGIT and LAG3 and exhaustion associated SOCS1-3 and CISH. Data obtained at time points of 1 month (1m) and 2 months (2m) after ex vivo isolation of hTERT and/or iCasp9 modified NK cells cultured with IL2+K562-mbIL21. 2-way Anova, Tukey's multiple comparisons, N1m=10, N2m=3, median, p-value: * $p < 0.05$; ** $p < 0.01$; *** $p < 0.001$.

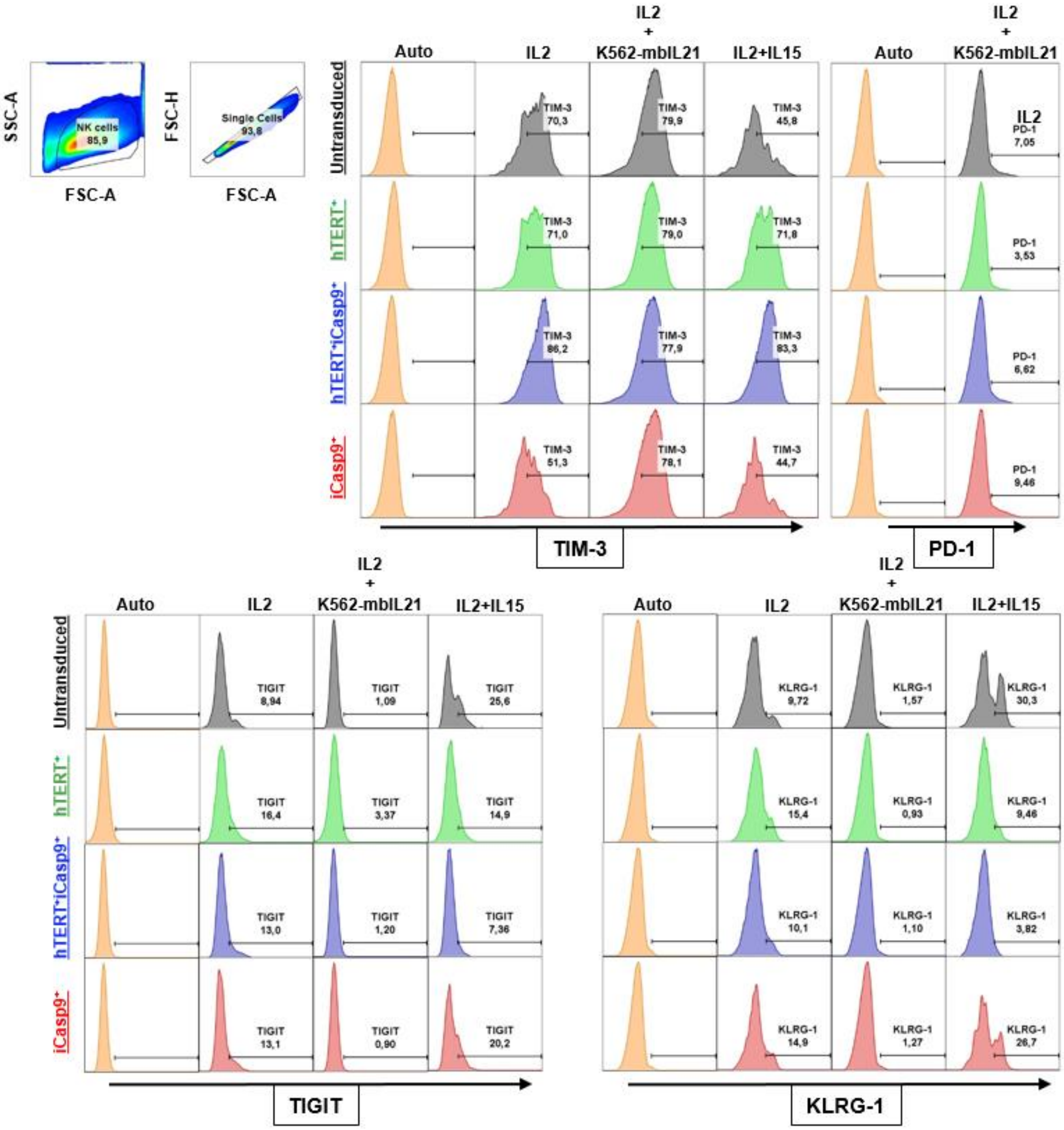

Figure S5. Representative plots describing gating strategy and representative fractions of TIM-3$^+$, TIGIT$^+$, KLRG-1$^+$ and PD-1$^+$ NK cells unmodified and modified with *hTERT* and/or *iCasp9* genes at a time point of 2 months after isolation. NK cells were cultured with 3 types of stimuli: IL2, IL2+K562-mbIL21, IL2+IL15.

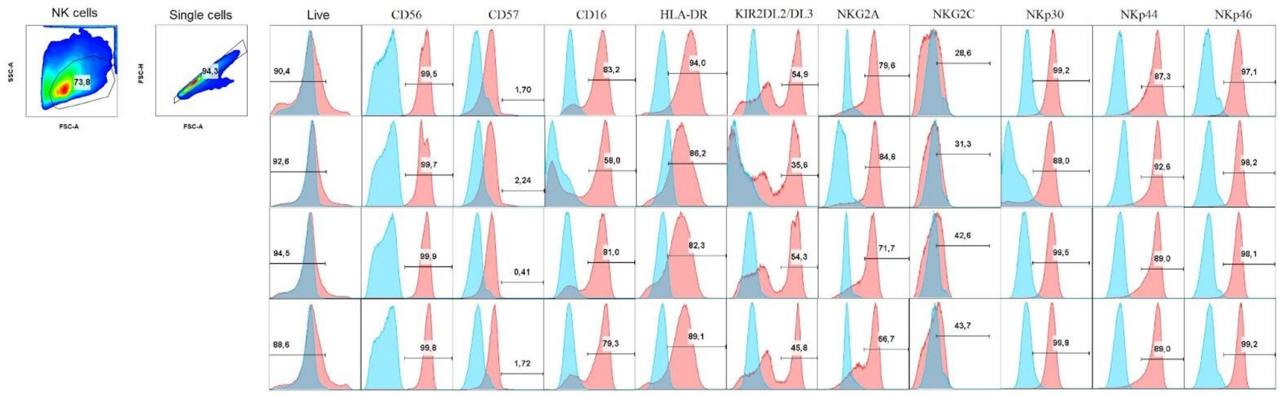

Figure S6. Representative plots describing gating strategy for NK cells, single cells and live cells and representative fractions for stained surface markers CD56, CD57, activating receptors CD16, HLA-DR, NKG2C, NKp30, NKp44, NKp46 and inhibitory receptors KIR2DL2/DL3 and NKG2A. Data presented for NK cells unmodified and modified with hTERT and/or iCasp9 genes at a time point of 2 months after isolation. NK cells were cultured with IL2+K562-mbIL21.

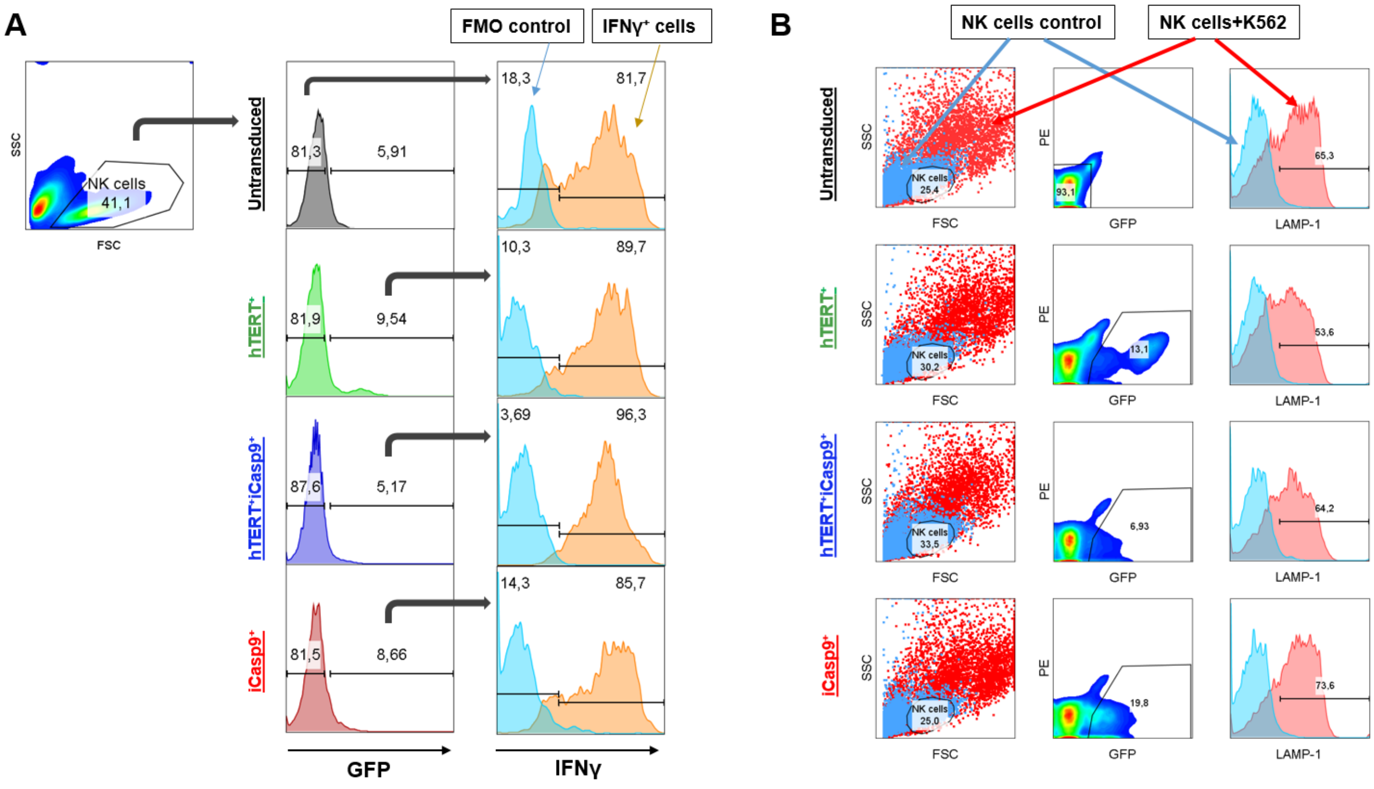

Figure S7. Representative plots describing gating strategy for NK cell functional assays. A) Cytokine (IL2, IL12, IL18)-induced IFNγ production. NK cells were divided on GFP- and GFP+ cells and that studied for IFNγ production. Light blue stands for FMO control. Orange histogram stands for NK cells stained with anti-INFγ antibodies. B) NK cell degranulation determined by LAMP-1 surface exposure in response to target cell (K562 cells) recognition. Blue corresponds with control NK cells incubated without K562 cells and red stands for NK cells mixed with targets.
